# Deepening our understanding of quality improvement in Europe (DUQuE): overview of a study of hospital quality management in seven countries

**DOI:** 10.1093/intqhc/mzu025

**Published:** 2014-03-25

**Authors:** Mariona Secanell, Oliver Groene, Onyebuchi A. Arah, Maria Andrée Lopez, Basia Kutryba, Holger Pfaff, Niek Klazinga, Cordula Wagner, Solvejg Kristensen, Paul Daniel Bartels, Pascal Garel, Charles Bruneau, Ana Escoval, Margarida França, Nuria Mora, Rosa Suñol, on behalf of the DUQuE Project Consortium, N Klazinga, DS Kringos, MA Lopez, M Secanell, R Sunol, P Vallejo, P Bartels, S Kristensen, P Michel, F Saillour-Glenisson, F Vlcek, M Car, S Jones, E Klaus, S Bottaro, P Garel, M Saluvan, C Bruneau, A Depaigne-Loth, C Shaw, A Hammer, O Ommen, H Pfaff, O Groene, D Botje, C Wagner, H Kutaj-Wasikowska, B Kutryba, A Escoval, A Lívio, M Eiras, M Franca, I Leite, F Almeman, H Kus, K Ozturk, R Mannion, OA Arah, A Chow, M DerSarkissian, CA Thompson, A Wang, A Thompson

**Affiliations:** 1 Avedis Donabedian Research Institute, Universitat Autònoma de Barcelona, Barcelona, Spain; 2 London School of Hygiene and Tropical Medicine, London, UK; 3 Department of Epidemiology, Fielding School of Public Health, University of California, Los Angeles (UCLA), Los Angeles, CA, USA; 4 UCLA Center for Health Policy Research, Los Angeles, CA, USA; 5 Polish Society for Quality Promotion in Health Care, Krakow, Poland; 6 Institute of Medical Sociology, Health Services Research and Rehabilitation Science, University of Cologne, Cologne, Germany; 7 Department of Public Health, Academic Medical Center, University of Amsterdam, Amsterdam, The Netherlands; 8 NIVEL Netherlands Institute for Health Services Research, Utrecht, The Netherlands; 9 Central Denmark Region & Center for Healthcare Improvements, Aalborg University, Aalborg, Denmark; 10 European Hospital and Healthcare Federation, Brussels, Belgium; 11 Haute Autorité de la Santé, Paris, France; 12 Portuguese Association for Hospital Development, Lisbon, Portugal; 13 Portuguese Society for Quality in Health Care, Porto, Portugal; 14 Red de Investigación en Servicios de Salud en Enfermedades Crónicas REDISSEC, Spain

**Keywords:** quality management systems, clinical indicators, clinical effectiveness, quality of healthcare, hospitals, cross-national research, patient outcomes

## Abstract

**Introduction and Objective:**

This paper provides an overview of the DUQuE (Deepening our Understanding of Quality Improvement in Europe) project, the first study across multiple countries of the European Union (EU) to assess relationships between quality management and patient outcomes at EU level. The paper describes the conceptual framework and methods applied, highlighting the novel features of this study.

**Design:**

DUQuE was designed as a multi-level cross-sectional study with data collection at hospital, pathway, professional and patient level in eight countries.

**Setting and Participants:**

We aimed to collect data for the assessment of hospital-wide constructs from up to 30 randomly selected hospitals in each country, and additional data at pathway and patient level in 12 of these 30.

**Main outcome measures:**

A comprehensive conceptual framework was developed to account for the multiple levels that influence hospital performance and patient outcomes. We assessed hospital-specific constructs (organizational culture and professional involvement), clinical pathway constructs (the organization of care processes for acute myocardial infarction, stroke, hip fracture and deliveries), patient-specific processes and outcomes (clinical effectiveness, patient safety and patient experience) and external constructs that could modify hospital quality (external assessment and perceived external pressure).

**Results:**

Data was gathered from 188 hospitals in 7 participating countries. The overall participation and response rate were between 75% and 100% for the assessed measures.

**Conclusions:**

This is the first study assessing relation between quality management and patient outcomes at EU level. The study involved a large number of respondents and achieved high response rates. This work will serve to develop guidance in how to assess quality management and makes recommendations on the best ways to improve quality in healthcare for hospital stakeholders, payers, researchers, and policy makers throughout the EU.

## Introduction

Research on quality in health care has built a sizeable knowledge base over the past three decades. Evidence on the effectiveness of organizational quality management and improvement systems has emerged more recently [1–4]. This recent research has addressed several questions. Do quality improvement activities lead to better quality of care? Which quality improvement tools are most effective? How can various quality tools be integrated into a sensitive and effective quality and safety improvement programme? What factors impact on the implementation of quality strategies at hospital level? [5, 6].

Recent European Union (EU) projects, such as the ‘Methods of Assessing Responses to Quality Improvement Strategies’ (MARQUIS) and the ‘European Research Network on Quality Management in Health Care’ (ENQUAL), have attempted to measure the effects of a variety of quality strategies in European hospitals in order to provide information that payers could use as they contract for the care of patients moving across borders. This prior research has had limitations including an incomplete conceptualization of antecedents and effects of quality management systems, methodological weaknesses in measurement strategies and limited use of data on patient outcomes in the evaluation of the effectiveness of these strategies [7–12].

This paper provides an overview of the conceptual framework and methodology of the recently completed DUQuE project (Deepening our Understanding of Quality Improvement in Europe) funded by the European Commission 7th Framework Programme. At its start, the goal of this project was to evaluate the extent to which organizational quality improvement systems, organizational culture, professional involvement and patient involvement in quality management are related to the quality of hospital care, assessed in terms of clinical effectiveness, patient safety and patient experience in a large and diverse sample of European hospitals.

DUQuE had the following specific objectives:

1. To develop and validate an index to assess the implementation of quality management systems across European hospitals (addressed in three papers by Wagner *et al*. [13–15]).
2. To investigate associations between the maturity of quality management systems and measures of organizational culture, professional engagement and patient involvement in quality management, including board involvement on quality and its association with quality management systems (addressed by Botje *et al*. [16]), the relationship between organizational culture and structure and quality management systems (addressed by Wagner *et al*. [17]) and the development and validation of the measures for evaluation of clinical management by physicians and nurses (addressed by Plochg *et al*. [18]).
3. To investigate associations between the maturity of quality management systems and patient level measures (PLMs) of clinical effectiveness, patient safety and patient experience including the relationships between quality management systems at hospital and pathway levels (addressed by Wagner *et al*. [19]), patient safety and evidence based management (addressed by Sunol *et al*. [20]), and patient involvement in quality management (addressed by Groene *et al*. [21]).
4. To identify factors influencing the uptake of quality management activities by hospitals, including external pressure as enforced by accreditation, certification or external assessment programmes (addressed by Shaw *et al*. [22]) and the feasibility of using routine data to compare hospital performance (addressed by Groene *et al*. [23]).

## Conceptual framework

**Figure 1:**
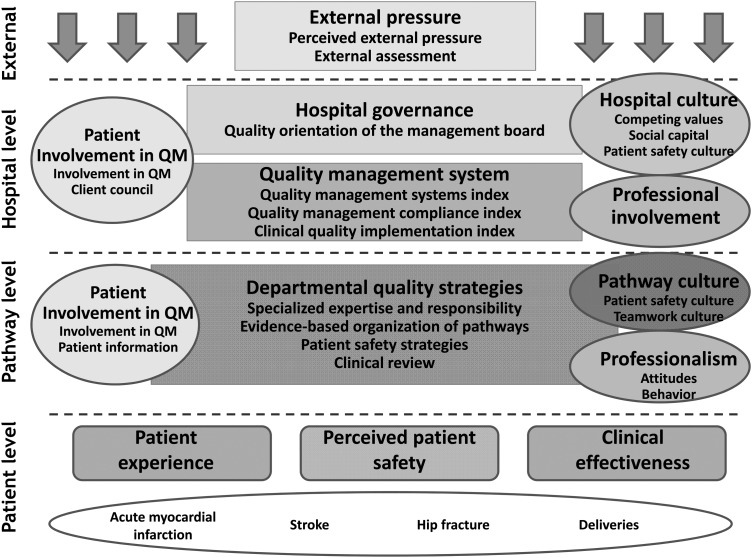
DUQuE conceptual framework.

The DUQuE study conceptual model was developed by a range of collaborators with a diverse range of disciplinary backgrounds and expertise. The model addresses four levels: hospital, departmental (or pathway) level, patient's level and external factors influencing uptake of management decisions (Fig. 1). We hypothesized that hospital level quality management systems would be associated with hospital governance and culture, the degree of professional involvement in management (and more concretely in quality management) and the degree of patient involvement in quality management. We further hypothesized that quality management systems in place to manage specific clinical pathways (sometimes referred to as ‘clinical service lines’) would be associated with quality management activities at the hospital and pathway level as well as with pathway culture, professionalism and patients' involvement in quality management. Moreover, we expected quality management systems at both hospital and clinical pathway levels to be associated with patients' experience, and the safety and clinical effectiveness of patient care for four selected conditions (acute myocardial infarction (AMI), obstetrical deliveries, hip fracture and stroke). These conditions were selected because they cover an important range of types of care, there are evidence-based standards for process of care against which compliance could be assessed and there is demonstrated variability in both compliance with process of care measures and outcomes of care (complications, mortality) that would allow for analysis of associations between these measured constructs. Finally, the conceptual model posited that factors external to these quality management systems would also influence the uptake of quality management activities such as perceived pressure from hospital leadership (chief executive officers and governance boards) and external accreditation, certification and standards programmes that offer audit and feedback regarding quality management and performance.

## Development of measures

For each construct in the conceptual model, published literature was searched to identify measure sets and instruments used previously. Explicit criteria were used to assess and select measures, including psychometric properties, level of evidence and appropriateness in multi-national studies. Whenever a validated measure, instrument or measure set existed and was deemed appropriate, we requested permission to use it in the DUQuE study. Where we found no existing measures or instruments, new measures were developed. After synthesis, 25 measure domains were defined and included in DUQuE (Table 1). Measures were originally designed in English language and translated to local languages when necessary. Table 2 shows the number of cases expected and finally gathered for each instrument designed to operationalize measures according to hospital type. All of the final instruments used are available at the DUQuE's website [24].

**Table 1:** Constructs, measure domains and data collection methods used

Construct name	Measure domain	Measure domain definition	Data collection method	Administration system
External pressure	Perceived external pressure	Influence in hospital management of external factors (accreditation, contracts, press …)	Questionnaire to CEO	Electronically administered questionnaire
	External assessment	Whether the hospital has undergone external assessment (accreditation, ISO)	Assessment at hospital level performed by an external visitor	Visit at hospital level performed by an external visitor. Both, paper and electronically administered audit forms
Hospital governance	Quality orientation of the management board	Including background in quality, time allocated for quality in the meetings, etc.	Questionnaire to CEO	Electronically administered questionnaire
Hospital level quality management systems (QMS)	Quality Management System Index (QMSI)	Index to assess the implementation of quality management system at hospital level	Questionnaire to hospital quality manager (QM)	Electronically administered questionnaire
	Quality management Compliance Index (QMCI)	Measuring compliance with quality management strategies to plan, monitor and improve the quality of care	Assessment at hospital level performed by an external visitor	Both paper and electronically administered audit forms
	Clinical Quality Implementation Index (CQII)	Index measuring to what extent efforts regarding key clinical quality areas are implemented across the hospital	Assessment at hospital level performed by an external visitor	Both paper and electronically administered audit forms
Hospital culture	Organizational culture Competing values framework (CVF)	CVF has two dimensions: structure of internal processes within the hospital and orientation of the hospital to the outside world	Questionnaires to Chair of Board of Trustees, CEO, Medical Director and the highest ranking Nurse	Electronically administered questionnaire
	Social capital	Measures common values and perceived mutual trust within the management Board	Questionnaire to CEO	Electronically administered questionnaire
	Hospital patient safety culture	Safety Attitude Questionnaire (SAQ): Two dimensions measuring perceptions on patient safety culture in terms of teamwork and safety climate	Questionnaires to leading physicians and nurses	Electronically administered questionnaire
Hospital professional involvement	Professional involvement in management	Measures leading doctors and nurses involvement in management, administration and budgeting and managing medical and nursing practice	Questionnaires to leading physicians and nurses	Electronically administered questionnaire
Patient involvement in quality management	Patient involvement in quality at hospital level	This construct assesses patients' involvement in setting standards, protocols and quality improvement projects. These constructs used in previous research (Groene, ENQUAL)	Questionnaire to hospital quality manager	Electronically administered questionnaire
	Client council	Measures existence and functioning of client council	Questionnaire to hospital quality manager	Electronically administered questionnaire
Department quality strategies	Specialized expertise and responsibility (SER)	Measures if specialized expertise and clear responsibilities are in place at pathway level	Assessment at pathway or department settings performed by an external visitor	Both paper and electronically administered audit forms
	Evidence-based organization of pathways (EBOP)	Measures if pathways are organized to deliver existing evidence base care	Assessment at pathway or Department settings performed by an external visitor	Both paper and electronically administered audit forms
	Patient safety strategies (PSS)	Measures if most recommended safety strategies are in place at ward level	Assessment at pathway or department settings performed by an external visitor	Both paper and electronically administered audit forms
	Clinical review (CR)	Measures if clinical reviews are performed systematically	Assessment at pathway or department settings performed by an external visitor	Both paper and electronically administered audit forms
Department pathway culture	Pathway patient safety culture	SAQ: two dimensions measuring perceptions on patient safety culture in terms of teamwork and safety climate	Questionnaires to physicians and nurses at pathway level	Questionnaire electronically administered
Professionalism	Professionalism	Measures professional attitudes towards professionalism and behaviour in their clinical area	Questionnaires to professionals at pathway level	Questionnaire electronically administered
Patient involvement in quality management	Patient involvement in quality at departmental level	This construct assesses patient's involvement in setting standards, protocols and quality improvement projects. These constructs used in previous research (Groene, ENQUAL)	Questionnaire to manager of care pathways or head of department	Questionnaire electronically administered
	Patient information strategies in departments	Measures if information literature, surveys and other activities are conducted at pathway or department level	Assessment at pathway or department settings performed by an external visitor	Both paper and electronically administered audit forms
Patient experience	Generic patient experience	Generic measure of patient experience (NORPEQ)	Patient survey	Paper-based questionnaire
	Perceived patient involvement	Measures perceived involvement of care (from Commonwealth Fund sicker patients survey)	Patient survey	Paper-based questionnaire
	Hospital recommendation	Measure of hospital recommendation (from HCAHPS)	Patient survey	Paper-based questionnaire
	Perceived continuity of care	Measures patient-perceived discharge preparation (Health Care Transition Measure)	Patient survey	Paper-based questionnaire
Perceived patients safety	Perceived patients safety	Measures patients' perception of possible harm and its management	Patient survey	Paper-based questionnaire
Clinical effectiveness	Clinical effectiveness indicators for AMI, stroke, hip fracture and deliveries	A set of clinical process composite indicators based on their high evidence of impacting patients' outcomes	Patient clinical charts Administrative hospital data	Electronic data collection sheet

**Table 2:** Data collection and response rates in the DUQuE project

Type of questionnaire	Expected *n* questionnaires per hospital with partial data collection	Expected *n* questionnaires per hospital with comprehensive data collection	Total questionnaires expected	Total questionnaires obtained	Average response rate	Response rate country range^a^
Professional questionnaires:			10 759	9857	90	78–98
A. Chair of the Board of Trustees	1	1				
B. Chief executive officer	1	1				
C. Chief medical officer	1	1				
D. Quality manager	1	1				
E. Leading physicians and nurses	20	20				
F. Manager of care pathways or head of department	0	4				
G. Professionals at pathway level	0	80				
M. Highest ranking nurse in the hospital	1	1				
Patient questionnaires	0	120	8670	6750	75	52–90
Chart reviews	0	140	10 115	9082	89	66–100
External visits	0	1	74	74	100	100–100
Administrative routine data	1	1	188	177	94	77–100
Total amount	26	371	29 806	25 940	87%	

^a^By country, % response rate range (minimum–maximum).

**Figure 2:**
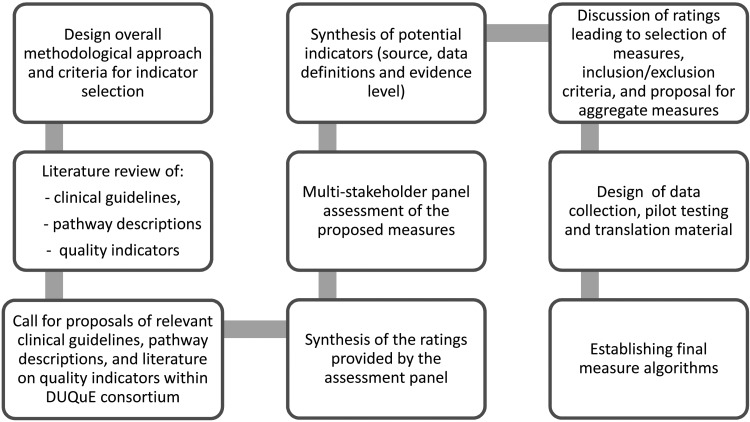
Development process of DUQuE clinical indicators.

The DUQuE project used a variety of measures to assess patient experience including a generic patient experience instrument (NORPEQ), a measure of patient-perceived discharge preparation (Health Care Transition Measure) and a single-item measure for perceived involvement of care and hospital recommendation in a DUQuE patient survey instrument. In addition, clinical indicators to assess key patient processes and outcomes were selected using a multi-step process (Fig. 2) that included a search of peer-reviewed literature and extensive searches of guidelines and other sources (a substantial amount of patient safety literature appears in the so-called ‘grey literature’ and not in peer-reviewed publications).

Clinical indicators for each of the four clinical conditions included in the study were collected, reviewed and rated based on the level of evidence according to the Oxford Centre of Evidence-Based Medicine [25] and their previous use in multi-national studies. In the next step, five relevant European scientific societies, five individual key experts with knowledge of the health-care systems in Europe and the eight DUQuE field test coordinators, named country coordinators, took part in a multi-stakeholder panel. Participants assessed the proposed clinical indicators using a rating matrix. Criteria applied to select four to six clinical indicators of process and outcome per condition were: comparability, data availability, data quality across the eight countries participating, relevance in the different EU health-care settings and ability of the PLM to distinguish between hospitals. The country coordinators offered structural and epidemiological information about treatment of each of the four clinical conditions. The final indicators were selected from among those with the highest level of evidence and the best multi-stakeholder panel rating results with previous use of an indicator in clinical cross-national comparisons given special consideration.

In each condition one or two composite measures were developed that combined the selected indicators for the purpose of a global analysis of the condition. Table 3 shows the final DUQuE indicator list with the source and level of evidence rating for each. Data from clinical indicators were collected via medical records abstraction using a standardized data collection method.

**Table 3:** Clinical indicators and composite measures selected for the DUQuE project

Condition	Main clinical indicators used	Source^a^	Level of evidence
Acute myocardial infarction (AMI)	Fibrinolytic agent administered within 75 min of hospital arrival	AHRQ	A
	Primary percutaneous coronary intervention within 90 min	AHRQ	A/B
	Thrombolytic therapy or primary percutaneous coronary intervention given	See 1a and 2a	See 1a and 2ª
	Therapy given on time^b^		
	Anti-platelet drug prescribed at discharge (ASPRIN)	AHRQ	A
	Beta blocker prescribed at discharge	AHRQ	A
	Medication with statin prescribed at discharge	AHRQ	A
	ACE inhibitors prescribed at discharge	AHRQ	A
	Appropriate medications: all four of anti-platelet, beta blocker, statin, ACE inhibitor) prescribed at discharge^b^		
Deliveries	Epidural anaesthesia applied within 1 h after being ordered for vaginal births	The Danish Clinical Registries	D
	Exclusive breastfeeding at discharge	WHO	D
	Blood transfusion during intended or realized vaginal birth	The Danish Clinical Registries	B
	Acute Caesarean section		
	Obstetric trauma (with instrumentation)	OECD	B
	Obstetric trauma (without instrumentation)	OECD	B
	Mother complication: unplanned C-section, blood transfusion, laceration and instrumentation^b^	The Danish Clinical Registries	**B**
	Adverse birth outcome (child)^b^	The Danish Clinical Registries	**B**
	Birth with complications^b^	The Danish Clinical Registries	**B**
Hip fracture	Prophylactic antibiotic treatment given within 1 h prior to surgical incision	RAND	A
	Prophylactic thromboembolic treatment received on the same day as admission (within 24 h or on same date when (one or more) times not provided)	RAND	A
	Early mobilization (within 24 h or before next day when (one or more) times not provided)	The Danish Clinical Registries	B
	In-hospital surgical waiting time <48 h [or 1 day when (one or more] times not provided)	OECD	C
	Percentage of recommended care per case: indicators prophylactic antibiotic treatment within 1 h, prophylactic thromboembolic treatment within 24 h, early mobilization within 24 h, in-hospital surgical waiting time <48 h = YES)^b^		
Stroke	Admitted to a specialized stroke unit within 1 day after admission	The Danish Clinical Registries	A
	Platelet inhibitor treatment within 2 days after admission	The Danish Clinical Registries	A
	Diagnostic examination within first 24 h/same day after admission using CT or MRI scan	The Danish Clinical Registries	D
	Mobilized within 48 h (or 2 days when times are missing) after admission?	The Danish Clinical Registries	C/D
	Appropriate stroke management. All three applied: Platelet inhibitor treatment within 2 days after admission, Diagnostic examination (CT or MRI) within first 24 h and Mobilized within 48 h^b^		

^a^According the Oxford Centre of Evidence-Based Medicine.

^b^Composite measures (aggregation of indicators).

## Design and fieldwork protocol

DUQuE was designed as a multi-level cross-sectional study with data collection in eight countries. Selected countries had to have a sufficient number of hospitals to fulfil sampling criteria, represent varied approaches to financing and organizing health care, have research staff with experience in conducting comprehensive field tests and represent the geographical reach of the EU. Turkey was included because of the status of its EU candidacy at the start of the project. The countries invited to participate in the field test were the Czech Republic, England, France, Germany, Poland, Portugal, Spain and Turkey.

### Sampling strategy

We aimed to collect data for the assessment of hospital-wide constructs from up to 30 randomly selected hospitals in each of the 8 participating countries (*N*_h_ = 240 hospitals, maximum). In each country, for 12 of these 30 hospitals we carried out comprehensive data collection at pathway and patient levels (*n*_h_ = 96 hospitals, maximum).

General acute care hospitals (public or private, teaching or non-teaching) with a minimum hospital size of 130 beds were considered for inclusion into the study. In addition, for comprehensive data collection, hospitals were required to have a sufficient volume of care to ensure recruitment of 30 patients per condition over a 4-month period (a sample frame of a minimum of 90 patients). Specialty hospitals, hospital units within larger hospitals, and hospitals not providing care for the four clinical conditions of study were excluded from further consideration.

Each country coordinator provided to the Central Project Coordination Team (CPCT) a de-identified hospital list (including all hospitals with more than 130 beds, ownership and teaching status) of each country for sampling purposes. A simple random sample was taken to identify candidate hospitals for comprehensive and partial data collection; this was oversampled to anticipate withdrawal of participants. The sampling strategy proved efficient as the distribution of key characteristics such as number of beds, ownership and teaching status did not differ between country-level samples and national distributions.

### Recruitment

After the preliminary sample of hospitals was selected, each country coordinator formally invited the hospital chief executive officers (CEOs) to participate. A total of 548 hospitals were approached of which 192 agreed to participate. The percentage of hospitals accepting participation ranged from 6.7 to 96.8% between countries. The main reasons of declining to participate were related to time constraints, organizational aspects and the complexity of the study. Data from 188 hospitals in 7 participating countries were included in the final analysis. After significant efforts, hospitals in England were not included partly due to delays in obtaining ethical approval and also extensive difficulty recruiting hospitals.

### Data collection

**Figure 3:**
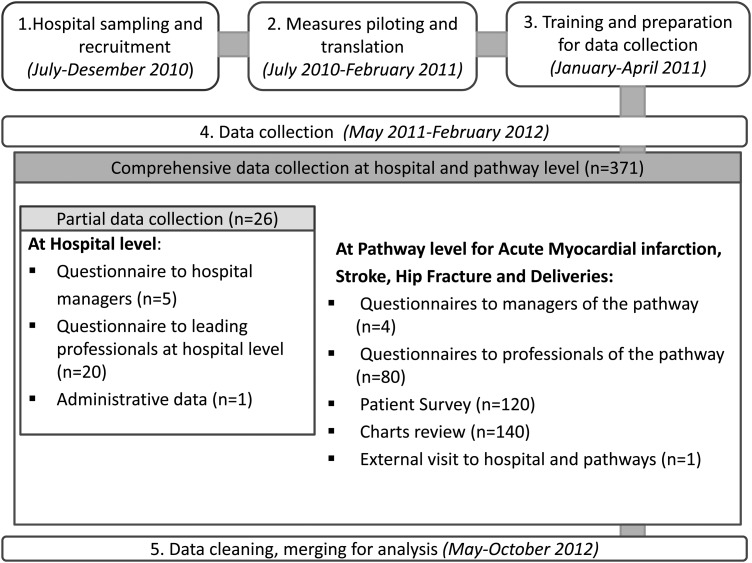
Stages of DUQuE field test.

The CPCT facilitated communication and problem solving during the ongoing field test process. Preparation began in July 2010 and included sampling, recruitment, back and forward translation and piloting of measures, preparation of materials and training hospital coordinators in charge of retrieving data. Data collection began formally in May 2011 and was completed by February 2012. Several coordination actions enabled timely data collection of professional questionnaires, patients' surveys, charts review, external visits and administrative data (Fig. 3).

An information technology platform was created to support central collection of responses to professional questionnaires and data from site visits. Among a subset of 88 hospitals a comprehensive data collection required them to provide administrative data and administer 371 questionnaires including patients, professionals, chart reviews and an external visit (Table 2). The remaining hospitals provided administrative data and administered professional questionnaires to 25 respondents. The overall response rate for the different questionnaires was between 75 and 100% for the assessed measures (Table 2).

Once field period was completed, all data collected was cross-checked by the CPCT to ensure that hospital and respondents’ codes matched per country. Data were also checked for any discrepancies, specifically the chart review files. All data sets were merged and transferred to the data analysis team.

### Ethical and confidentiality

DUQuE fulfilled the requirements for research projects as described in the 7th framework of EU DG Research [26]. Data collection in each country complied with confidentiality requirements according to national legislation or standards of practice of that country.

## Discussion

The DUQuE project has achieved several important advances that are detailed in the papers included in this supplement. First, it is the first large-scale quantitative study exploring across a broad and diverse sample of European hospitals several features of the implementation of hospital quality management systems and their impact on quality and safety. It represents a major advance over previous research by including patient-level processes and outcomes, enabling study of the impact of current quality improvement strategies on patient care and outcomes. Secondly, it made use of a comprehensive conceptual model describing the functioning of hospital quality management systems and supporting a deliberate empirical, quantitative measurement and analytic approach. Thirdly, it succeeded in collecting and linking large amounts of data from hospitals in seven countries including professional surveys (management and baseline), patient surveys, clinical indicators from medical records and administrative data from participating hospitals. Fourthly, the DUQuE coordination structure and protocol including periodic follow-up and feedback to hospital coordinators enabled the project to obtain the active participation of hospitals and high response rates. Finally, the project led to the development of new measures, reported in this supplement, which can be used in future research.

Despite these many achievements, the study has limitations that are worth highlighting. First, the study was not designed or powered to report on the results for each country. We pooled data across countries and addressed heterogeneity in country-level estimates in our statistical modelling using an approach that allowed country-level baselines and effects to vary. Further covariate adjustment included hospital size, ownership and teaching status. A second limitation is that we used a cross-sectional study design. This was considered most appropriate for hypothesis testing in this project because it requires a relatively shorter time commitment and fewer resources to conduct, although ultimately does not allow strong conclusions about causality [27]. The use of directed-acyclic graphs guided the development of our statistical models, incorporating theory and knowledge derived from previous research findings and this approach helps somewhat in strengthening causal inferences from the project results. A third limitation is related to the sampling strategy. Although a random sampling strategy was used, a generalization to participating countries and hospitals is limited because hospitals volunteered to participate in the project introducing potential self-selection bias. However, our analysis shows substantial variations in the implementation of quality criteria, and outcomes, suggesting that the effect of self-selection may be less problematic. A final limitation is the existence of systematic biases due to self-report and to country differences in data coding. The study relied on self-reported data that may introduce social desirability bias. In many instances, we were able to use other data sources to verify responses, but not for all measures. Measuring clinical practice using clinical record review may introduce systemic biases related to medical record completeness practice variations among countries, but some of this is handled through statistical adjustment for the effects of country, hospital size, ownership and teaching status. In conclusion, while the design is prone to a number of limitations we anticipated potential problems and addressed them in study design and our analytical strategy.

This supplement presents the first set of results from the DUQuE project. It includes quality management indexes conceptualization and development, validation of new measures and results of tests of associations between some of the main constructs. Future publications are intended to address remaining questions and secondary analyses of this very large database. For example, papers addressing the associations between quality management systems and key patient processes and outcomes are planned. In addition to the research publications, the DUQuE project website offers a set of recommendations presented in an appraisal scheme derived from the broader perspective of this investigation and summarized for hospital managers buyers and policy makers who may find it useful as they redesign organization-wide approaches to hospital care to achieve the highest levels of quality and safety [24].

## Funding

This work was supported by the European Commission's Seventh Framework Program FP7/2007–2013 under the grant agreement number [241822]. Funding to pay the Open Access publication charges for this article was provided by European Community's Seventh Framework Programme (FP7/2007–2013) under grant agreement no. 241822.

## Appendix

The DUQuE project consortium comprises: Klazinga N, Kringos DS, MJMH Lombarts and Plochg T (Academic Medical Centre-AMC, University of Amsterdam, THE NETHERLANDS); Lopez MA, Secanell M, Sunol R and Vallejo P (Avedis Donabedian University Institute-Universitat Autónoma de Barcelona FAD. Red de investigación en servicios de salud en enfermedades crónicas REDISSEC, SPAIN); Bartels P and Kristensen S (Central Denmark Region & Center for Healthcare Improvements, Aalborg University, DENMARK); Michel P and Saillour-Glenisson F (Comité de la Coordination de l'Evaluation Clinique et de la Qualité en Aquitaine, FRANCE) ; Vlcek F (Czech Accreditation Committee, CZECH REPUBLIC); Car M, Jones S and Klaus E (Dr Foster Intelligence-DFI, UK); Bottaro S and Garel P (European Hospital and Healthcare Federation-HOPE, BELGIUM); Saluvan M (Hacettepe University, TURKEY); Bruneau C and Depaigne-Loth A (Haute Autorité de la Santé-HAS, FRANCE); Shaw C (University of New South Wales, Australia); Hammer A, Ommen O and Pfaff H (Institute of Medical Sociology, Health Services Research and Rehabilitation Science, University of Cologne-IMVR, GERMANY); Groene O (London School of Hygiene and Tropical Medicine, UK); Botje D and Wagner C (The Netherlands Institute for Health Services Research-NIVEL, the NETHERLANDS); Kutaj-Wasikowska H and Kutryba B (Polish Society for Quality Promotion in Health Care-TPJ, POLAND); Escoval A and Lívio A (Portuguese Association for Hospital Development-APDH, PORTUGAL) and Eiras M, Franca M and Leite I (Portuguese Society for Quality in Health Care-SPQS, PORTUGAL); Almeman F, Kus H and Ozturk K (Turkish Society for Quality Improvement in Healthcare-SKID, TURKEY); Mannion R (University of Birmingham, UK); Arah OA, Chow A, DerSarkissian M, Thompson CA and Wang A (University of California, Los Angeles-UCLA, USA); Thompson A (University of Edinburgh, UK).
